# Prevalence of self-stigma and its association with self-esteem among psychiatric patients in a Nepalese teaching hospital: a cross-sectional study

**DOI:** 10.1186/s12888-019-2344-8

**Published:** 2019-11-07

**Authors:** Shanta Maharjan, Bimala Panthee

**Affiliations:** Patan Academy of Health Sciences, School of Nursing and Midwifery, Lalitpur, Nepal

**Keywords:** Mental illness, Self-esteem, Self-stigma

## Abstract

**Background:**

Stigma against mental illness cuts across all age, religion, ethnic origin or socio-economic status. Similarly, self-stigma among psychiatric patients is also prevalent worldwide. The consequences of self-stigma are low self-esteem, increased severity of symptoms, low treatment adherence, increased rate of suicidality and decreased quality of life. Thus, this study aims to find the prevalence of self-stigma and its association with self-esteem of patients with mental illness in Nepal.

**Methods:**

This was a cross-sectional study conducted among 180 patients with mental illness attending a psychiatric Outpatient Department (OPD). Non-probability purposive sampling technique was used for the study. The data was collected by face to face interview technique. Structured interview schedule questionnaire (brief version of internalized stigma scale and Rosenberg self-esteem scale) was used to collect the data. Descriptive statistics, inferential statistics and correlation analysis were used for data analysis. *P* value was set at 0.05.

**Results:**

Overall prevalence of self-stigma was 54.44%. Among those who had self-stigma 48% had mild self-stigma, 34.7% had moderate self-stigma and 17.3% had severe self-stigma. Among the five components of self-stigma scale, the highest mean score was on stereotype endorsement, followed by discrimination experience, social withdrawal, stigma resistance, and the lowest for the component of alienation. Furthermore, strong negative correlation (*r* = − 0.74) was found between self-stigma and self-esteem. The correlation was still significant (*r* = − 0.69) after controlling for socio-demographic and clinical variables. Hospital admission and diagnostic category of respondents were significantly associated with self-stigma. However, no significant association was found between socio-demographic variables and self-stigma.

**Conclusion:**

Based on the findings of this study, it can be concluded that self-stigma is prevalent among psychiatric patients in Nepal. Most of the respondents experienced stereotype endorsement. Also, higher self-stigma is significantly associated with poor self-esteem suggesting self-stigma reduction programs. Furthermore, strong negative relationship between self-stigma and self-esteem suggests some causal relationship studies to confirm if self-esteem enhancement program can be beneficial to reduce self-stigma among psychiatric patients.

## Background

Globally, mental health problems are alarming issue accounting for 13.3% of Disability Adjusted Life Years (DALY), and 32.4% of all Years Lived with Disability (YLD) [1]. Mental disorders are accompanied by social reactions that add a dimension of suffering, also called “second illness” or “stigma” [2]. Stigma against mental illness cut across all age, religion, ethnic origin or socio-economic status [3].

Most of the literatures highlight the existence of two dimensions of stigma namely, public stigma and self-stigma or internalized stigma [4]. Public stigma is the negative attitudes held by members of the public about devalued people. Whereas self-stigma occurs when people internalize those public attitudes and suffer numerous negative consequences as a result [4].

Prevalence of self- stigma among psychiatric patients is high ranging from 22.5 to 97.4% in different countries. For instance, it was 36% in USA [5], 97.4% in Ethiopia [6], 22.5% in Nigeria [7], 49.5% in China [8] and 50–66% in India [9]. Furthermore, self-stigma among psychiatric patients is associated with poor quality of life [10], low treatment adherence [11], decreased self-esteem [12, 13], increase in severity of symptoms, low level of self-efficacy and poor recovery [12]. In extreme conditions, self-stigma is associated with the higher rate of suicidality [14].

Nepal is not an exception towards stigma for mental illness, where, public stigma towards mental illness was 43.6% [15]. Public stigma was measured using devaluation discrimination scale developed by Link and colleagues. In this study public stigma was assessed among students of Nepal it can be related to cultural aspects of Nepal. It is known that public stigma leads to self-stigma [16, 17]. However, to our knowledge, self-stigma among mental ill people has not been studied till date in Nepal. Thus high public stigma towards mental illness might have been exaggarating the self-stigma of psychiatric patients in Nepal. Similarly, self-stigma is strongly associated with low self-esteem that is directly related to the prognosis and complication of the disease condition. On the other hand, positive self-esteem is basic feature of mental health that protects the people from impact of negative influences of mental illness. Furthermore, it facilitates for effective coping. Thus self-esteem acts as a protective factor in mental health [18]. This vital element of mental health is threatened by the self-stigma among psychiatric patients.

The prevalence and disease burden of mental disorders is incredibly high in Nepal. It is estimated that 18% of the non-communicable disease (NCD) burden is due to mental illness. Using the global estimates, approximately 1% Nepalese may be affected with severe mental disorders while 10–20% people have one or other minor mental health problems [19]. However, availability of mental health services is different from other countries where only one governmental mental hospital is providing mental health services in the country. Also, going to mental hospital for the mental health services is the matter of shame for many Nepalese people. Likewise, Nepal is a multi-cultural country where social factors play a vital role in determination of mental health of people.

Thus this study aimed: (i) to identify the prevalence of self-stigma of psychiatric patients in Nepal (ii) to examine the relationship between self-stigma and self-esteem among psychiatric patients (iii) to examine the factors (socio-demographic and clinical variables) associated with self-stigma of psychiatric patients. Based on the previous research results, we hypothesized that self-stigma would be negatively associated with self-esteem and socio-demographic and clinical variables would be associated with self-stigma. Our study will contribute to improve quality of life of Nepalese people with mental illness. These findings would indicate the importance of considering self-stigma and self-esteem to support Nepalese people who suffer from mental illness.

The concept of the study was guided by modified labeling theory developed by Link et al. [20]. Modified labeling theory explains how the self-identity and behavior of individuals are determined or influenced by the terms or label used to describe or classify them. This theory was used because the concept of labeling used in this theory could justify the process of self-stigma. It also explains the negative outcomes of stigma on self-esteem.

The evidence of hazardous effect of self-stigma on self-esteem in psychiatric patients calls the need for the program to reduce self-stigma. Furthermore, an understanding of stigma and discrimination experienced by this group is vital to develop appropriate training materials. Especially, for low-income countries it needs more detailed understanding of the extent and nature of the stigma related to mental illness.

## Methods

### Design

A cross-sectional design was used for the study. It was conducted in Psychiatric OPD of Patan Hospital (a teaching hospital) located in capital city of Nepal. It is one of the largest teaching hospital in Nepal.

### Participants

One hundred eighty psychiatric patients attending psychiatric OPD during 1 month period of data collection (12th August to 7th September, 2018) were purposively included in the study. Psychiatric patients who had insight were included in the study. In this study, insight refers to degree of awareness the patient has about one’s own illness. Insight was assessed using the scale given by Saddock in 2009 [21]. Insight was assessed because without being aware of own’s illness or condition, patient might not feel that he/ she is being stigmatized. Moreover, to understand the statements in ISMI scale and RSES, the patient needed conscious understanding of his condition. Also, those who were willing to participate and who gave consent to participate in the study were included. The patients with any substance related disorders or who were violent or diagnosed with organic mental disorders were excluded from the study. The patient with substance abuse and organic mental disorders were excluded from the study because of the potential impact of recent substance abuse on the experience of stigma and degree of awareness.

### Measures

Structured interview schedule was developed for collection of data. The interview schedule had following parts: (1) Questions related to socio demographic and clinical variables (2) Brief version of Internalized stigma of mental illness scale (ISMI-10) to assess self-stigma and (3) Rosenberg Self Esteem Scale (RSES) to assess self-esteem.

#### Socio demographic variables and clinical variables

Socio-demographic variables included age, gender, area of residence, education, type of family, status of marriage, employment status and clinical variables included duration of illness, co-morbidity, family history of mental illness, hospital admission and diagnosis. Hospital admission means if the respondents had any history of hospital admission because of the psychiatric illness before in dichotomous option yes/ no. If the respondents have history of hospital admission because of psychiatric illness, they were further asked about how many times they were admitted in number of hospital admissions in open ended options as continuous variable.

#### Brief version of internalized stigma of mental illness scale (ISMI-10)

ISMI-10 was developed by Boyd and Otilingam in 2014 in USA [2]. It is a 10-item 4 point Likert scale, where 1 indicates strongly disagree and 4 indicates strongly agree. It shows good content and criterion validity. The sensitivity was 0.87 and the specificity was 0.94. The reliability was also in acceptable range i.e. 0.75. In this study the reliability was maintained by utilizing Cronbach alpha which was 0.74. Interpretation of score was done by following the method used by Lysaker et al. [22]. Two items (item 2 and 9) were reversed before calculating the total score. After that, all the item scores were added and then divided by the total number of answered items. The resulting score ranged from 1 to 4. For example, if someone answers 8 of the 10 item, the total score is produced by adding together the 8 answered items and dividing by 8.

Stigma was categorized into 4 levels. For instance; score 1.00–2.00 = no to minimal self-stigma, 2.01–2.50 = mild self-stigma, 2.51–3.00 = moderate self-stigma, and 3.01–4.00 = severe self-stigma.

#### Rosenberg self esteem scale (RSES)

RSES was developed by Rosenberg in 1965 [23]. It is a 10-item scale. The scale measures state self-esteem by asking the respondents to reflect on their current feelings. The original sample for which the scale was developed consisted of 5024 high-school juniors and seniors from 10 randomly selected schools in New York State. The scale generally has high reliability: test-retest correlations are typically in the range of 0.82 to 0.88, and Cronbach’s alpha for various samples are in the range of 0.77 to 0.8. In this study Cronbach’s alpha for this scale was 0.78. Total score ranges from 10 to 40. Higher score was interpreted as higher self-esteem [16].

Both questionnaire (ISMI-10 and RSES) were translated into Nepali utilizing the guideline by Wild et al. [24]. Also, socio-demographic and clinical variables were translated into Nepali language. Face validity was done after finalization of translation. No modification was needed. Nepali version of the interview schedule was used for the study.

### Data collection procedure

Data was collected among the patients who visited OPD confirming the diagnosis and insight of the patients. Insight was assessed by the psychiatrist and researcher. Patient’s chart was used to confirm the type of illness (diagnosis). After selection of the patient, the objectives of the study were well explained to the patient and verbal informed consent was taken from each patient. Data was collected by using face to face interview technique where researcher read the questions aloud to the participants. Though the tool used were self-report measure, face to face interview was done utilizing interview schedule because many respondents were illiterate. Also, those who could read might interpret the statement differently. Thus, to bring homogeneity in the understanding of the statements, it was read to all respondents. Nine patients were interviewed in a day for 5 working days in a week.

### Statistical analysis

All the collected data were checked for completeness, consistency and accuracy. Data was organized, coded and entered in SPSS version 16.0 for analysis. The analysis and interpretation were done based on the objectives. For instance; descriptive statistics was used for demographic and clinical variables, Chi square test was used for examining factors associated with self-stigma and correlation and partial correlation analysis were used to examine relationship between self-stigma and self-esteem. *P* value was set at 0.05.

## Results

Mean age of respondents was 35 ± 12 years. Most of the respondents (80%) were between the age 20 and 50 years. More than half (61 and 60%) were married and unemployed, respectively. Regarding education 51% were illiterate.

Similarly, mean duration of illness was 4.2 ± 4.9 years. One fourth of the respondents had family history of mental illness. More than half (66%) of respondents did not have co-morbid conditions. Likewise, most of the patients (43.89%) had mood disorders (Table 1).

**Table 1:** Clinical variables of the respondents

		*N* = 180
Variables	Frequency	Percent
Duration of Mental Illness (in years) Mean ± SD 4.13 ± 4.98		
Less than 1	56	31.11
1 to 5	79	43.89
6 to 10	34	18.89
11 to 15	5	2.78
16 and above	6	3.33
Presence of Co-morbidity		
Yes	61	33.9
No	119	66.1
Family History of Mental Illness		
Yes	45	25.00
No	135	75.00
Hospital Admission		
Yes	57	31.67
No	123	68.33
Diagnostic Category		
Mood disorders	79	43.89
Neurotic, stress-related & somatoform disorders	72	40.00
Schizophrenia, schizotypal & delusional disorders	28	15.56
Disorders of adult personality & behavior	1	0.56

The prevalence of self-stigma was 54.4%. Among those who had self-stigma (*n* = 98) 48% had mild self-stigma, 34.7% had moderate self-stigma and 17.3% had severe self-stigma (Table 2). In terms of mean score on various domains of ISMIS the highest score was noted for stereotype endorsement (mean score = 3.62) followed by discrimination experience (mean score = 3.37) and social withdrawal (mean score = 3.31) (Table 3). Regarding correlation between self-stigma and self-esteem, there was significant negative correlation (*r* = −.74) (Table 4). The significant association was still present (*r* = −.69) after controlling for demographic and clinical variables (age, education, duration of illness and no. of hospital admission).

**Table 2:** Level of self-stigma among psychiatric patients attending in psychiatric OPD

			*N* = 98
Level of Stigma		Frequency	Percent
Mild self-stigma	(2.01–2.50)	47	48.00
Moderate self-stigma	(2.51–3.00)	34	34.70
Severe self-stigma	(3.01–4.00)	17	17.30

**Table 3:** Mean score and standard deviation of each sub-scale of ISMI-10

		*N* = 180
Sub-scale of ISMI-10	Mean	Standard Deviation
Alienation	2.36	0.86
Stereotype endorsement	3.62	1.11
Discrimination experience	3.37	1.29
Social withdrawal	3.31	1.39
Stigma resistance	2.86	1.01

**Table 4:** Zero order correlation between demographic variables, clinical variables, self-stigma and self-esteem

					*N* = 180	
Variables	Variables					
	Age	Education	Duration of illness	No. of hospital admissions	Self-Stigma	Self-esteem
Age	1.					
Education	−.41^a^	1				
Duration of illness	0.36^a^	−0.21^b^	1			
Number of hospital admissions	0.18	−0.37^b^	0.35^a^	1		
Self-stigma	−0.04	0.08	0.12	0.10	1	
Self-esteem	0.01	0.02	−0.06	0.03	−0.74^a^	1

^a^Correlation is significant at the 0.01 level (2-tailed)

^b^Correlation is significant at the 0.05 level (2-tailed)

Regarding the factors associated with self-stigma, none of the socio-demographic variables (gender, area of residence, educational status, type of family, marital status, present status of marriage, employment status) were significantly associated with self-stigma. But, significant association was found between clinical variables and self-stigma. History of hospital admission (χ^2^ = 5.93, *p* = 0.01) and diagnostic category (χ^2^ = 8.57, *p* = 0.01) were significantly associated with self-stigma. It means that, those patients who had history of hospital admissions had more stigma than those who had not been admitted in hospital. Similarly, respondents with mood disorders and schizophrenia had more self-stigma. But there was no significant association between co-morbidity, family history of mental Illness and self-stigma (Table 5).

**Table 5:** Association between clinical variables and level of self-stigma

				*N* = 180
Clinical Variables	Minimal to Mild Self-stigma n (%)	Moderate to Severe Self-stigma n(%)	χ^2^ value	*P*-value
Presence of Comorbid Conditions				
Yes	42(68.85)	19(31.15)	0.36	0.55
No	87(73.11)	32(26.89)		
Family History of Mental Illness				
Yes	33(73.33)	12(26.67)	0.08	0.77
No	96(71.11)	39(28.89)		
Hospital admission due to mental illness				
Yes	34(59.65)	23(40.35)	5.93	0.01
No	95(77.24)	28(22.76)		
Diagnostic category				
Schizophrenia, schizotypal & delusional disorders	18(64.29)	10(35.71)	8.57	0.01
Mood disorders	50(63.29)	29(36.71)		
Neurotic, stress-related & somatoform disorders, Disorders of adult personality & behavior	61(83.56)	12(16.44)		

## Discussion

The first objective of this study was to find the prevalence of the self-stigma. Overall prevalence of self-stigma was 54.44%. Among those who had self-stigma, 48% had mild self-stigma, 37.3% had moderate stigma and 17.7% had severe self-stigma. This prevalence rate was similar to the cross-sectional study done in India where the prevalence rate was 50–66% among patients of schizophrenia and Alcohol Dependent Syndrome (ADS) [9]. Resemblances in social structures and culture may be the reason for similar rates of prevalence in Nepal and India. But a study conducted in Ethiopia showed contrasting result where the prevalence rate was very high (97.4%) [6]. Another study in Korea showed low prevalence rate (8.1%) [25]. Differences in diagnostic category of patients in each study might be the reason for this variation high and low level of self-stigma. For example, the above mentioned study in Ethiopia included patients with schizophrenia and the study in Korea included most of the patients with bipolar disorders. As 55% of Nepalese psychiatric patients reported self-stigma, it highlights the need for mental health providers to develop strategies and tools to minimize self-stigma, and to develop interventions for its prevention and amelioration.

For the components of stigma-scale, the mean score was highest for stereotype endorsement, followed by discrimination experience, social withdrawal, stigma resistance, and the lowest for the component of alienation. Higher score for stereotype endorsement in Nepalese patients reflects more negative societal attitude towards patients with mental illness. And lower rates of alienation (which reflects isolation and shame) can be understood in the socio-cultural context. In Nepal, most of the families live on joint family thus they can take care their ill person. This possibly leads to lower alienation. Similar findings were found in India [26, 27]. However, studies conducted in India are focused on patients with schizophrenia and the patients in remission. But this study included most of respondents (84%) with mood disorders and neurotic disorders (Table 1). Due to these differences in diagnostic category, all the findings of the above mentioned Indian study cannot be generalized in this study.

The second objective of the study was to examine the relationship between self-stigma and self-esteem. Our findings showed strong negative relationship between self-stigma and self-esteem (*r* = − 0.74). The strong negative relationship was present even after controlling for demographic and clinical variables. It means that as the level of self-stigma increases the self-esteem of psychiatric patient decreases. The causality cannot be determined here, though. When self-esteem is decreased it directly affects the treatment of disease process. It may lead to low treatment adherence [11] and also lead to suicide in severe cases [14]. Similar finding (*r* = 0.71) was found in a study conducted in 2013 in Israel among patients with schizophrenia [28]. But, inconsistent result was found in another study done in India which demonstrated that self-stigma was not correlated with self-esteem [29].

Third objective of the study was to identify the factors associated with self-stigma. None of the socio-demographic variables (age, gender, area of residence, education, type of family, marital status, employment status) were significantly associated with self-stigma. This result is consistent with the findings of systematic review done in 2010 [30], where, none of the socio-demographic variables (gender, age, education, marital status, employment status, living arrangement, and ethnicity) were significantly associated with self-stigma. However, in another study, self-stigma was different according to gender where, female patients had higher self-stigma than male patients [31]. Likewise, self-stigma was positively correlated with level of education and employment in a study in China. Self-stigma was found higher in those who were educated and employed [32]. Similarly, the patients living jointly with family had higher self-stigma (*p* < 0.05) than those living alone in a study done in Japan among 110 patients with depression [33]. Furthermore, unmarried patients had high self-stigma [6]. Though different studies found the difference in level of self-stigma according to socio-demographic variables, we did not find difference in our study. It is suggested to confirm this results utilizing large scale study and focusing on homogenous sample of psychiatric patients.

Regarding clinical variables of the patient, hospital admission and diagnostic category were significantly associated with self-stigma. More self-stigma was seen among those who have been admitted in hospital. This might be because those who have been admitted have to deal with hospital staffs, other people in hospital and society. They might face negative public attitudes during their contact with those people of society. Internalization of such public attitudes might have caused them to develop self-stigma. Higher self-stigma associated with admission might also mean that there is higher symptom severity, which was not measured in this study. A study done in 2015 showed contrasting result which showed no relationship between hospital admission and self-stigma [34]. Likewise, another study done in USA showed no significant relationship between hospital admission and self-stigma [5].

There was variation in level of self-stigma according to the diagnostic category of the patient. The patient with mood disorder (36.71%) and schizophrenia (35.71%) had more self-stigma than those with neurotic disorders (16.44%). It might be because of differences in severity of signs and symptoms, the differences in duration of illness and differences in period of treatment according to the diagnosis or type of illness. This result is consistent with a study done in 2017 where, it was found that people with schizophrenia reported more experiences of discrimination as a result of stigma than those with PTSD [35]. While, duration of mental illness had weak positive relationship with self-stigma(*r* = 0.12) which means as the duration of illness increases the level of self-stigma also increases. Those people, who have been ill for a long period, have lived more years with psychiatric disability. Experience of psychiatric symptoms for many years and experience of negative public attitude for many years in this type of patient might have increased self-stigma. This finding is similar to the study done in 2013 where duration of illness was positively associated with self-stigma [25]. However, previous study found no significant correlation between duration of illness and self-stigma [27]. Other clinical variables like co-morbidity and family history of mental illness did not have significant relationship with self-stigma. These findings are similar to the findings of previous studies [36].

## Limitations of the study

There are some limitations of this study. First, duration for data collection was limited. Though calculated sample were 316, only 180 samples could be included in the study. Second, the study was focused on self-stigma of psychiatric patients of various types of psychiatric illness. More specific studies could be conducted focusing on specific psychiatric illness, so that self-stigma status in particular illness can be determined. Also, we studied only self-esteem as a consequence of self-stigma. There are other constructs that self-stigma could affect. Likewise, omitting patients with histories of substance abuse or violence might have affected the levels of self-stigma reported in this study as these groups of people may experience quite a bit high stigma. Though the tool used were self-report measure it was read aloud to the participants as most of them were illiterate and could not understand the meaning of statements that might have influence the level of self-stigma to answer the responses to the researcher. We also could not explain the causality relationship in this study. However, our study has some strength. Though we included only one hospital, it is one of the biggest teaching hospital where all kinds of patients come for check-up from different areas of the country. Thus, patients who came to visit this hospital can reflect the general population of the country.

## Conclusion

Self-stigma was prevalent in more than half of psychiatric patients. Most of the respondents experienced stereotype endorsement suggesting the need of self-stigma reduction strategies in community level in Nepal. Also, strong negative relationship between self-stigma and self-esteem suggests some causal relationship studies to confirm if self-esteem enhancement program can be beneficial to reduce self-stigma among psychiatric patients.

Implementation of awareness program could help to reduce self-stigma. Furthermore, self-stigma and self-esteem had strong negative relationship. It suggests that as self-stigma increases self-esteem decreases. So to protect self-esteem of psychiatric patient self-stigma must be reduced. Different interventions targeting self-stigma like healthy self-concept, self-stigma reduction program, ending self-stigma, and cognitive therapy might help in reducing self-stigma [37]. Also, self-esteem enhancement programs can decrease self-stigma. Likewise, those patients who were admitted in hospital had significantly high self-stigma than those who were not admitted. Negative attitudes of public (hospital staffs, other people in society) they face during the process of admission, might have caused them to internalize such negative attitudes leading to self-stigma. So, it suggests for reduction of public stigma as well.

## Data Availability

The data set used and/or analyzed during the current study will be available from the corresponding author on reasonable request. Individual responses will not be shared.

## Abbreviations

DALY: Disability Adjusted Life Year
IRC: Institutional Review Committee
ISMI-10: Internalized Stigma of Mental illness Inventory–10-item Version
OPD: Outpatient Department
PAHS: Patan Academy of Health Sciences
RSES: Rosenberg Self-esteem Scale
SPSS: Statistical Package for Social Science
YLD: Years Lived with Disability
